# Experimental and FLUKA simulation study of CdO /Al_2_O_3_ cement waste marble composites for nuclear radiation shielding

**DOI:** 10.1038/s41598-025-21061-y

**Published:** 2025-10-08

**Authors:** Ahmed M. El-Khatib, Mahmoud I. Abbas, Malak H. Eid, M. Fayez-Hassan, Mona M. Gouda

**Affiliations:** 1 Physics Department, Faculty of Science, Alexandria University, Alexandria, 21511 Egypt; 2 Experimental Nuclear Physics Department, Nuclear Research Center, Egyptian Atomic Energy Authority, Cairo, Egypt

**Keywords:** Cement composite, Waste marble, Building materials, Micro-CdO, Nano-CdO, Micro-Al_2_O_3_, Nano-Al_2_O_3_, LAC, MAC, HVL, MFP, TVL, EABF, Mechanical, SEM, TEM, FLUKA simulation, Gamma and neutron shielding, Nanoscience and technology, Physics

## Abstract

Nuclear Radiation shielding materials are essential in various industries, especially nuclear power, medical imaging, and space exploration. This research delves into the exploration of radiation shielding efficacy by infusing cement and waste marble composites with micro and nanoparticles of Cadmium Oxide (CdO) and Aluminum Oxide (Al_2_O_3_), focusing on key parameters such as half value layer (HVL), tenth value layer (TVL), linear attenuation coefficient (LAC), and mass attenuation coefficient (MAC). These composites show potential in enhancing the shielding performance due to the unique properties of the nanoparticles. We systematically characterize the structural, morphological, and radiation-shielding properties of the composites through experimentation. In addition to the Absorption buildup factor (EABF) was held in this study to help in enhancing the shielding material as it is an important parameter. The outcomes demonstrated that the CdO-Al_2_O_3_ particle addition enhanced the composites’ radiation shielding capabilities. Raising the weight% (Wt.%) of CdO-Al_2_O_3_ particles improved the efficiency of the shield. The outcomes also showed that nano-sized CdO-Al_2_O_3_ particles outperformed micro-sized particles in radiation shielding. The work shows the potential of CdO- Al_2_O_3_ doped cement-waste Marble matrix composites for applications including shielding against gamma radiation. This study correspondingly investigates the shielding capabilities using the FLUKA Monte Carlo code. The simulation employed a wide range of photon and neutron energies, up to 100 and 20 MeV respectively. The results show the effectiveness of the introduced composite in attenuating both gamma rays and neutrons, highlighting their potential applications in radiation shielding.

**Supplementary Information:**

The online version contains supplementary material available at 10.1038/s41598-025-21061-y.

## Introduction

Several jurisdictions of the world are exploring the use of nuclear technology in place of fossil fuels. Given the growing importance of radioisotopes in various industrial and medical fields, research into the absorption potential of common building materials such as clay, rocks, and concrete are necessary^1–8^. Depending on the application, Various materials are often utilized for this purpose. For instance, concrete is typically used to line the walls of X-ray rooms because it is practical and effectively absorbs X-rays. Although concrete is generally a good option, other materials are sometimes necessary, as concrete can crack and lose water after extended exposure to radiation^9^.

The finest materials for absorbing radiation doses are those with high density and heavy metals like barium and lead. Ionizing radiation can have hazardous effects, thus people have employed lead and lead compounds as a kind of protection^10^.Because lead is inexpensive and may be used to shield radiation from radiation, it is commonly used in medical applications for nuclear medicine, X-ray, and equipment containers to safeguard the safety of both technicians and patients^11,12^. Unfortunately, lead is poisonous despite its beneficial properties^13^. and fails to have suitable mechanical attributes. Many materials and composites have had their shielding effectiveness examined, and their linear attenuation coefficients (LAC) have been found^14,15^.

The new material offers a cost-effective, safe substitute for traditional, ecologically dangerous products. The majority of recent studies on novel materials have been on composites consisting of several building materials and high-density metals^16^. Sustainable materials are becoming more and more important, with the preference being given to using waste paper, plastic, glass^17,18^, and metal as raw materials rather than disposing of it in landfills. This lowers the extraction of raw materials and minimizes waste and pollution^19^. Recycled aggregates have drawn a lot of attention as a potential use in new mortar and concrete formulations. As alternatives to conventional aggregates, researchers have looked into a variety of recycled aggregates, such as waste glass^18^, coconut fiber^20^, iron slag^21^, PET plastic waste^22^, waste marble^23,24^, and bottom ash^25^.

Marble may be found in many places, such as on walls, floors, furniture, home goods, and antiques. Massive quantities of marble trash are produced by manufacturers, who contaminate the surrounding ecosystems of animals and agriculture with irregular stones and powder. This marble dust can be used to concrete in place of conventional natural coarse aggregate^26^.

Aluminum has a remarkable ability to withstand radiation damage; in fact, it can withstand radiation 100 times more effectively than materials normally used in spacecraft. As a radiation shield and structural enclosure, its remarkable strength-to-weight ratio and lightweight construction have established it as a mainstay of space gear. To slow down the impact of radiation particles, modern spacecraft use many layers of thin aluminum shields with air gaps in between. Moreover, aluminum is essential for building outer spacesuits, which protect astronauts from radiation exposure in the wide emptiness of space. Interestingly, nuclear protective suits are also made with aluminum radiation shielding. Personalized 3D printing is becoming more and more popular in the medical profession^27^. These shields are designed to provide cancer cells with significant radiation exposure during radiation treatment while minimizing the harm encountered by healthy tissues. This novel strategy has enormous potential to improve the efficacy of cancer treatment while lowering the risk of damage to healthy tissues^28^.

Cadmium oxide (CdO) poses serious risks to the environment because of its intrinsic toxicity and possible negative effects. However, in high-energy photon beam radiation treatment (E > 10 MeV), photons interact with high atomic number materials in the linac head and the beam collimation system to form interactions known as (γ,n), which mostly produces neutrons in the linac head. These neutrons influence the shielding requirements that must be met in radiation treatment rooms. They also raise the radiation dosage that patients receiving high-energy photon beam radiation therapy get out-of-field. Cd will aid in the absorption of these neutrons, particularly the heat ones^29^.

This study examined the radiation-shielding capabilities of mortar made from discarded marble and locally sourced cement, which is recycled from industries in an environmentally responsible manner. Additionally, marbles are a construction material that can be added to concrete mixtures in certain amounts to raise their density and boost gamma-ray attenuation. They may also be employed in radiation protection applications.

More research on the impact of filler size on the shielding characteristics against gamma radiation for various composite systems is highly desired. Therefore, the primary goal of this work is to examine how the weight% and particle size of CdO-Al_2_O_3_ particles affect their capacity to protect against gamma radiation.

A scanning electron microscope (SEM) was used to characterize the composites made using the compression cement molding process in order to confirm the size effect. After a practical evaluation of the mass attenuation coefficients of pure cement-waste marble mortar, six additional samples with varying weight percentages and particle sizes were made and assessed using a NaI scintillation detector at photon energies between 59.53 keV and 1323.01 keV. Furthermore, a comparative analysis of particle size’s capacity to protect against radiation was conducted.

Since nanoparticles have incredibly small intermolecular distances between molecules, increasing the probability of photon collisions with the material atoms, their ability to attenuate photons has improved recently. Due to its prospective qualities, which include their lightweight design and advantageous mechanical, chemical, and physical qualities^30,31^, Scientists are interested in how nanomaterials are being used in many areas of science and technology. The usage of nanoparticles as fillers in bulding matrices has been shown to be developing^32^. Several researches demonstrated the enhanced protective capability of cement composites by using nanoparticles as fillers in biulding materials. Metal- or heavy-element oxide-containing nanocomposites are highly valued by nuclear engineers. El-Khatib et al.^6^, for example, Impact of micro/nano cadmium oxide on shielding properties of cement–ball clay matrix and found that CdO nanoparticles are more effective at shielding against gamma radiation than micro-CdO particles. Thus, the focus of their research has been on developing these nanocomposites as a substitute for traditional radiation shielding.

For simulations the FLUKA Monte Carlo code is a widely used tool for simulating radiation transport and interaction processes. Its accurate physics models and comprehensive cross-section libraries, such as ENDF, enable reliable predictions of radiation attenuation in various materials. FLUKA finds applications in diverse fields including shielding design, radiation detector response studies, medical physics, and dosimetry calculations. This study utilizes FLUKA to evaluate the shielding capabilities of the introduced composites against both photons and neutrons. The simulations were performed using FLUKA code, coupled with its advanced graphical user interface FLAIR. Mono-energetic gamma rays (0.1 keV-100 MeV) and neutrons (from thermal to twenty MeV) were used to analysis the shielding properties of CdO/Al_2_O_3_ cement and waste marble composites, with Nano and Micro Particle Size, defined by their elemental composition. The simulation results, obtained through analysis of output binary files, provide valuable insights into the attenuation behavior of these materials.

## Materials and methods

### Materials

Firstly, Materials used in this investigation included Portland cement (supplied locally) and additionally, powdered waste marble collected from marble factories was dried, ground by a mechanical grinder, and then sieved to be used as an aggregate.

Secondly, micro-scale metal oxide (CdO) was purchased from the El-Gomhouria Company in Egypt. The average particle size of these oxides ranged from 50 to 100 μm, and their purity was up to 99%. Meanwhile, Nano-scale cadmium oxide (CdO) particles (average size 40 nm) were purchased from the Nanotech company in Egypt, where they were chemically prepared, As for Aluminum oxide (Al_2_O_3_) micro-scale was purchased from LOBA Chemie company India with purity (99%), and the Nano scale was Synthesis powder was produced by high-energy planetary ball milling (Fritsch Pulverisette 7, Fritsch, Weimar, Germany) at a rate of 500 rounds per minute (rpm). The ball mill contains four vials of size 50 ml made from tungsten carbide. Balls of different Nano iron slag powder was produced by high-energy planetary ball milling (Fritsch Pulverisette 7, Fritsch, Weimar, Germany) at a rate of 500 rounds per minute (rpm). The ball mill contains four vials of size 50 ml made from tungsten carbide. Balls of different sizes with a total mass of 90 g and a diameter between 2 and 10 mm were employed in the milling process, where the ball-to-powder weight ratio was set to be 5:1.

The size of the nano CdO and Al_2_O_3_ was analyzed using a transmission electron microscope (FE-TEM) manufactured by JEOL, Japan, operating at 200 kV. Furthermore, the samples were coated with an ultrathin gold coating using a low-vacuum sputtering coating device (JEOL-JFC-1100E), so The SEM images were obtained at magnification order of 5,000x at 20 kV. The scanning electron microscope (SEM) was employed to observe the distribution of micro- and nano-sized particles within the composites’ cross-section.

**Table 1:** Specimen codes and weight fraction in percentage (wt%) of cement, Marble, and CdO + AL_2_O_3_.

Weight fraction in percentage (wt%)	Cement (wt%)	Marble (wt%)	Al_2_O_3_ (wt%)	CdO (wt%)	Code
Micro	50	50	0	0	CM
	50	30	10	10	CM-M1
	50	20	15	15	CM-M2
	50	10	20	20	CM-M3
Nano	50	30	10	10	CM-N1
	50	20	15	15	CM-N2
	50	10	20	20	CM-N3

### Preparation of cement/marble/CdO/ Al_2_O_3_ composites

**Fig. 1:**
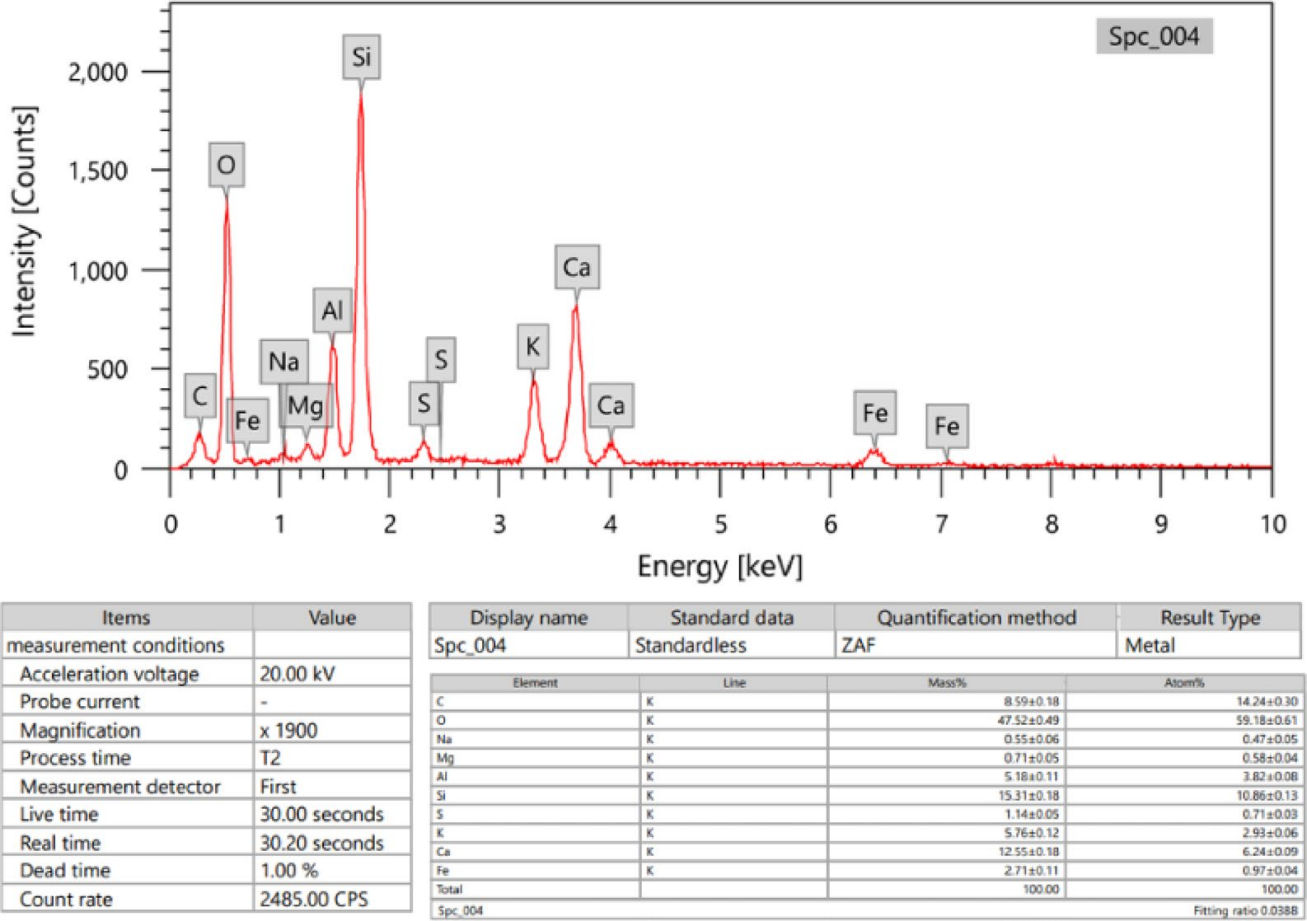
EDX spectra cement.

**Fig. 2:**
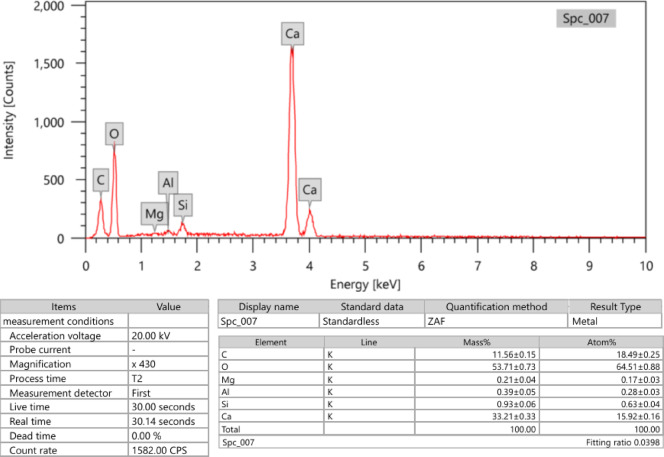
EDX spectra waste marble.

In order to create a homogenous mixture, the cement and marble were combined with the oxide quantities listed in Table 1 and their chemical composition was determined by energy dispersive x-ray (EDX) analysis showed in Figs. 1 and 2. The mixture was then thoroughly mixed using a mixer. The prepared ratio of cement to marble and metal-oxides are listed in Table 1 for the seven samples, with the coding that will be used throughout the study.

This powder combination shown in Table 2 which would be added to a mixture of water (mixture: water = 3:1) to make the compound, was delicately weighed with an electrical balance (Analytical Balance, GR200, Japan) with an accuracy of 0.0001 g. It was then placed in a plastic container and given two weeks to dry. Discs with a diameter of approximately 3 cm and a height of 2 cm have been made for the seven prepared samples.

All samples are made of same shape to match the specimens, and during their measurement they were placed 15 cm away from the source and 5 cm away from the crystal of the detector at room temperature.

**Table 2:** The composite sample designations and the weight fraction% of filler in each composite (EDX).

Elements	CM (wt%)	CM-M1 (wt%)	CM-M2 (wt%)	CM-M3 (wt%)
Ca	0.1957	0.1629	0.1465	0.1301
O	0.5365	0.4579	0.4186	0.3793
Si	0.0962	0.0952	0.0948	0.0943
Al	0.0543	0.1166	0.1477	0.1788
S	0.0044	0.0044	0.0044	0.0044
Fe	0.0125	0.0125	0.0125	0.0125
Mg	0.0039	0.0035	0.0034	0.0032
Ti	0.0037	0.0037	0.0037	0.0037
C	0.0928	0.0557	0.0371	0.0186
Cd	0.0000	0.0875	0.1313	0.1751

### Instrumentation

#### Gamma ray spectroscopy setup

The hermetically sealed Canberra U.S.A. Sodium Iodide Scintillation Detector, model number 802, has an aluminum shell, an internal magnetic light shield, a photomultiplier tube, a high resolution NaI (Tl) crystal, and a 14-pin connector. The NaI (Tl) detectors of the 802 series, available in both well and cylindrical shapes, provide excellent efficiency and reliable response. There is a history of consistency and long-term reliability with these detectors. The Model 2007Tube Base connects to any Model 802 assembly to power any Model 802 assembly. The Model 2007P tube base/preamplifier combination is also compatible with the Model 802. We used a NaI (Tl) detector (802-3 × 3in) with a 7.5% resolution at the Cs-137 peak at 661.66 in our study^33,34^.

**Fig. 3:**
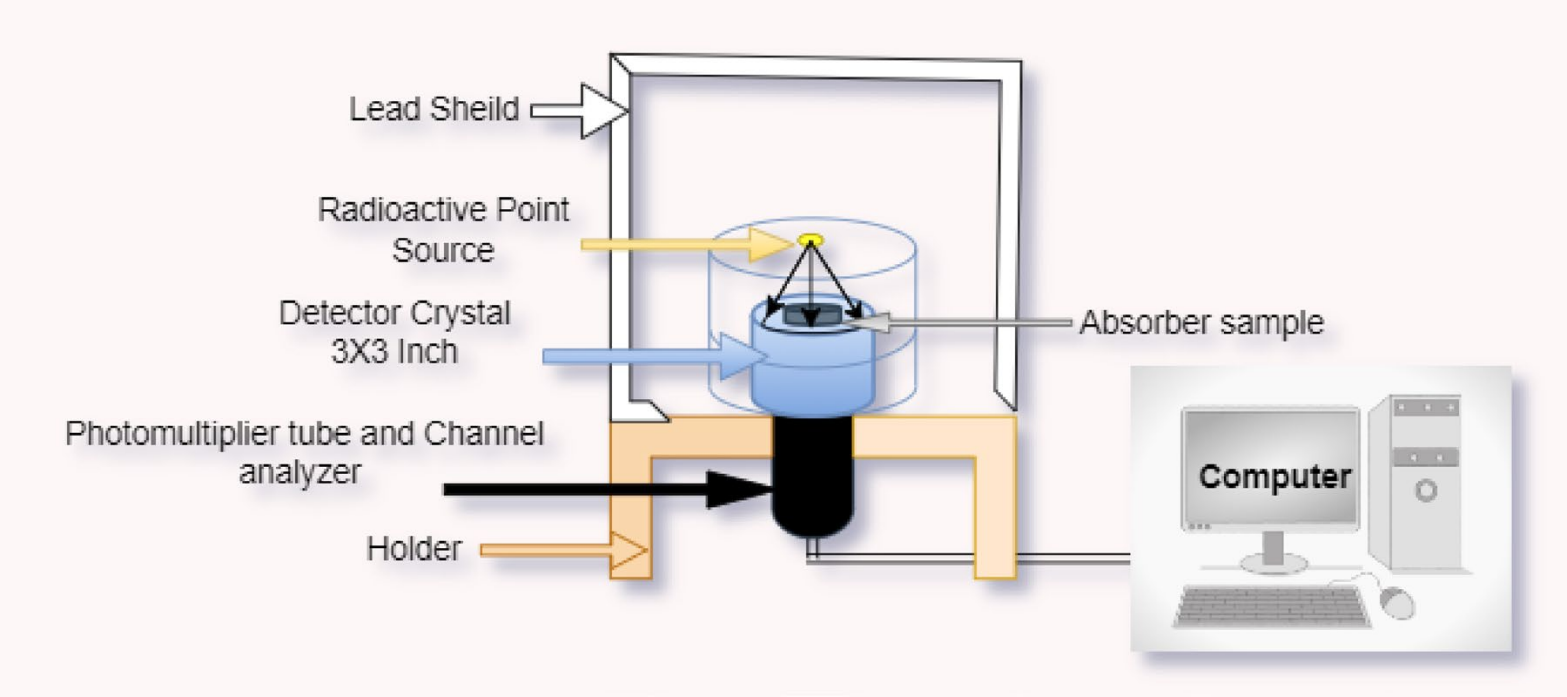
Experimental adjustment for gamma-ray measurement.

We used a NaI (Tl) detector (802-3 × 3in) at 661.66 keV, which has a resolution of 7.5% at the peak of Cs-137 in our study. In the energy range of 59.53 keV to 1332 keV, four typical radioactive point sources (Am-241, Ba-133, Cs-137 and Co-60) were used to monitor gamma radiation. The initial activity of these sources was 259, 275.3, 385, and 212.1 kBq. The current emission levels of these radioactive sources are 254.06, 125, 292.12 and 43.78 kBq in that order. To minimize measurement errors caused by detector dead time and provide a narrow beam, the radioactive source was positioned at a height of 5 cm throughout the experiment. Based on sample thickness, all measurements’ gamma spectra were gathered often enough to ensure a statistical error of less than 1%. The acquired spectra were analyzed using the Genie 2000 software. An Excel document was used to tabulate the net area beneath each peak in the spectrum at a particular energy and thickness to determine the shielding characteristics of the composites that were made. Figure 3 shows the experimental setup for the gamma measuring instrument.

#### Theoretical background

The count rate was computed both with and without the sample in Eq. (1)

1 $$\:\text{I}=\frac{\text{A}}{\text{t}}\:$$

Time to attain an error of less than or equal to 1% is denoted by t, where A is the area under the curve. The chance of photons interacting with matter per unit route length is known as the µ linear attenuation coefficient, or LAC ($\:\text{c}{\text{m}}^{-1}$), and it was empirically determined using the well-known Beer–Lambert law Eq. (2)^35^:

2 $$\:{\upmu\:}=\frac{1}{\text{x}}\text{ln}\left(\frac{{\text{I}}_{0}}{\text{I}}\right)$$

Where $\:{\text{I}}_{0}\:$and $\:\text{I}$ are the incident and transmitted intensities, respectively, passing through a target material of thickness x. The mass attenuation coefficient or MAC (µ/ρ) can be calculated by dividing the experimental linear attenuation coefficient (µ) of a given sample by its density (ρ) shown in Eq. (3)^36^,

3 $$\:\text{M}\text{A}\text{C}=\frac{{\upmu\:}}{{\uprho\:}}\:$$

and may be computed hypothetically using the NIST X-COM web application^16^.

Other important shielding parameters as half value layer (HVL) and tenth value layer (TVL) represent the thickness needed to attenuate 50% and 90% of the initial photon intensity, respectively, and can be evaluated by the following Eqs. (4) and (5) respectively^37^.

4 $$\:\text{H}\text{V}\text{L}=\frac{\text{L}\text{n}\left(2\right)}{{\upmu\:}}\:$$

5 $$\:\text{T}\text{V}\text{L}=\frac{\text{L}\text{n}\left(10\right)}{{\upmu\:}}\:$$

Moreover, the mean distance a photon travels in an absorber before being removed from the original beam by absorption or scattering is known as its mean free path (MFP). Furthermore, the shielding material’s highly significant attribute that is determined by Eqs. (6),

6 $$\:\text{M}\text{F}\text{P}=\frac{1}{{\upmu\:}}$$

Effective atomic number has also been calculated using Eq. (7)^38^:

7 $$\:{\text{z}}_{\text{e}\text{f}\text{f}}=\frac{\sum\:_{\text{i}}{\text{f}}_{\text{i}}{\text{A}}_{\text{i}}{\left(\frac{{\upmu\:}}{{\uprho\:}}\right)}_{\text{i}}}{\sum\:_{\text{i}}{\frac{{\text{A}}_{\text{i}}}{{\text{z}}_{\text{i}}}\left(\frac{{\upmu\:}}{{\uprho\:}}\right)}_{\text{i}}}\:\:$$

where $\:{\text{f}}_{\text{i}}$, $\:{\text{Z}}_{\text{i}}$ and $\:{\text{A}}_{\text{i}}$, refer to the molar fraction, atomic number, and atomic weight of the ith constituent element in the selected polymer, respectively.

When choosing a shielding material, the buildup factor must be considered to edit the absorption calculations resulting from buildup of secondary photons resulting from Compton scattering^39^. To determine the EBF for the selected polymers, the Geometric-Progression fitting method (GP) was employed, and the computations were determined according to the three following steps.

1. The absorption and exposure build up factors are from the significant parameters of for the shielding material that will be calculated by using the geometric progression (GP) fitting method carried out in three steps:
  First calculate equivalent atomic number of the composite, using the following Eq. (8)^40^:
  8 $$\:{\text{Z}}_{\text{e}\text{q}}=\frac{{\text{z}}_{1}\left(\text{l}\text{o}\text{g}{\text{R}}_{2}-\text{l}\text{o}\text{g}\text{R}\right)+{\text{z}}_{2}(\text{l}\text{o}\text{g}\text{R}-\text{l}\text{o}\text{g}{\text{R}}_{1})}{\text{l}\text{o}\text{g}{\text{R}}_{2}-\text{l}\text{o}\text{g}{\text{R}}_{1}}$$
  where $\:{\text{R}}_{1}$ and $\:{\text{R}}_{2}$ are the $\:{({\upmu\:}}_{\text{c}\text{o}\text{m}\text{p}}/{{\upmu\:}}_{\text{t}\text{o}\text{t}\text{a}\text{l}})\:$ratios corresponding to the elements with atomic numbers $\:{\text{Z}}_{1}\text{a}\text{n}\text{d}\:{\text{Z}}_{2}$ respectively, and R is the $\:{({\upmu\:}}_{\text{c}\text{o}\text{m}\text{p}}/{{\upmu\:}}_{\text{t}\text{o}\text{t}\text{a}\text{l}})\:$ratio for the investigated composites at a specific energy, which lies between ratios$\:{\:\text{R}}_{1}$ and $\:{\text{R}}_{2}$.
2. Second step after obtaining $\:{\text{Z}}_{\text{e}\text{q}}$ values for the composites and by using the GP fitting buildup factors coefficients (a, b, c, d,$\:{\:\text{x}}_{\text{K}})$ in the energies from 0.015 to 15 MeV using formula, Eq. (9)^41^:
  9 $$\:\text{C}=\frac{{\text{C}}_{1}\left(\text{l}\text{o}\text{g}{\text{Z}}_{2}-\text{l}\text{o}\text{g}{\text{Z}}_{\text{e}\text{q}}\right)+{\text{C}}_{2}\left(\text{l}\text{o}\text{g}{\text{Z}}_{\text{e}\text{q}}-\text{l}\text{o}\text{g}{\text{Z}}_{1}\right)}{\text{log}{\text{Z}}_{2}-\text{l}\text{o}\text{g}{\text{Z}}_{1}}\:$$
  where $\:{\text{C}}_{1}$ and $\:{\text{C}}_{2}$ are GP fitting parameters, taken from ANSI/ANS-6.4.3 standard database^42^, corresponding to the atomic numbers $\:{\text{Z}}_{1}$and $\:{\text{Z}}_{2}$ between which $\:{\text{Z}}_{\text{e}\text{q}}$ of the prepared composite.
3. Third and final step is to calculate the buildup factor using the obtained GP fitting parameters coefficient as follows in Eq. (10):
  10 $$\:\text{K}\left(\text{E},\text{x}\right)=\text{C}{\text{x}}^{\text{a}}+\text{d}\frac{\text{tanh}\left(\frac{\text{x}}{{\text{x}}_{\text{k}}-2}\right)-\text{tanh}\left(-2\right)}{1-\text{tanh}\left(-2\right)}\:,\:\text{f}\text{o}\text{r}\:\text{x}\le\:40\:\text{M}\text{F}\text{P}$$
  Where E is incident photon energy and X is the penetration depth in terms of MFP.
  So, the calculated build up factor is represented in Eqs. (11)and (12) and were calculated^42^:
  11 $$\:\text{B}\left(\text{E},\text{x}\right)=1+\frac{\text{b}-1}{\text{K}-1}\left({\text{K}}^{\text{x}}-1\right),\text{K}\ne\:1\:$$
  12 $$\:\:\text{B}\left(\text{E},\text{x}\right)=1+\left(\text{b}-1\right)\text{x},\text{K}=1$$

## Result and discussion

### Field emission-transmission electron microscope (FE-TEM)

**Fig. 4:**
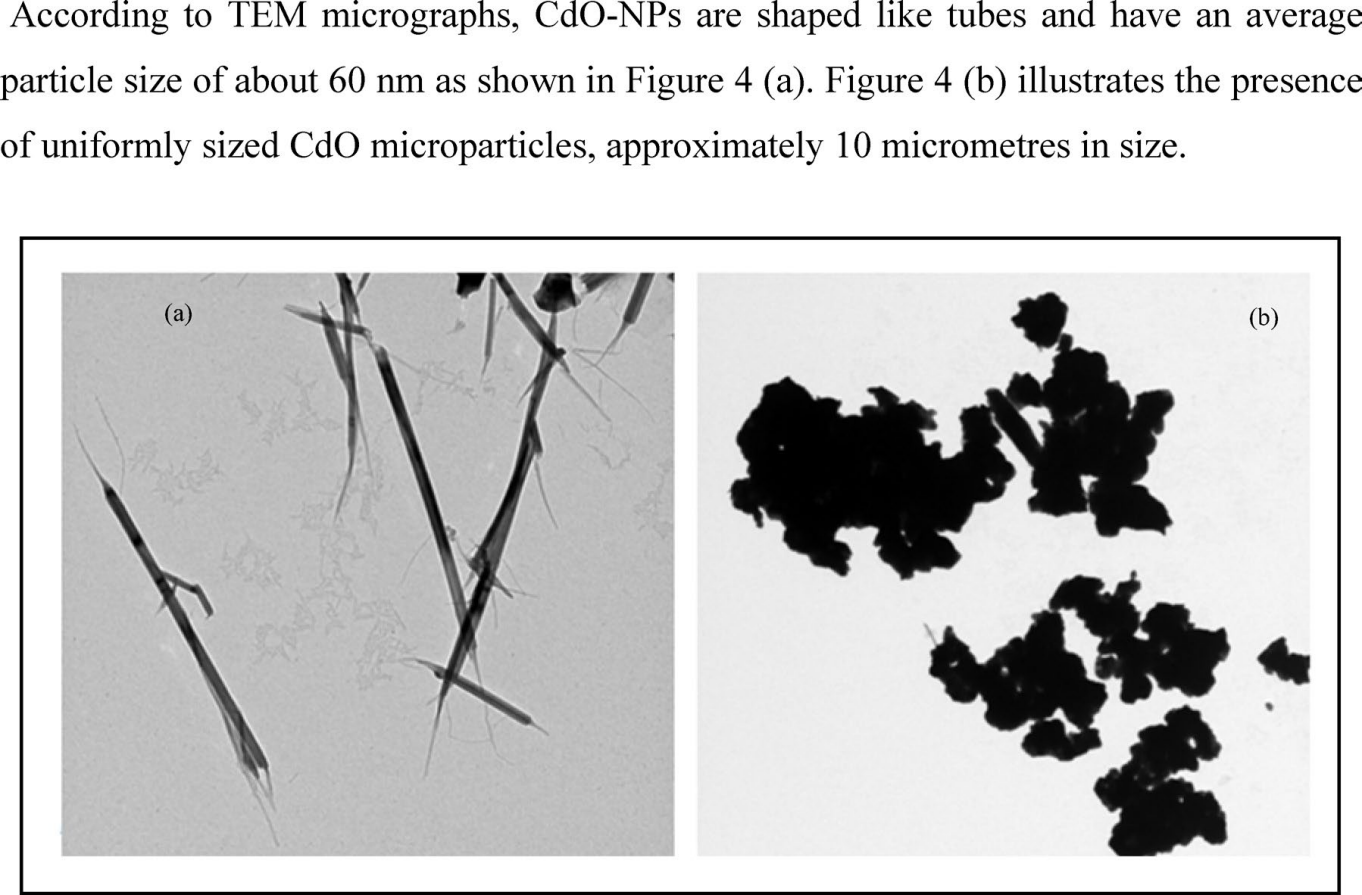
TEM images of (**a**) CdO nanoparticles size and (**b**) CdO microparticles size.

According to TEM micrographs, CdO-NPs are shaped like tubes and have an average particle size of about 60 nm as shown in Fig. 4a. Figure 4b illustrates the presence of uniformly sized CdO microparticles, approximately 10 micrometres in size.

**Fig. 5:**
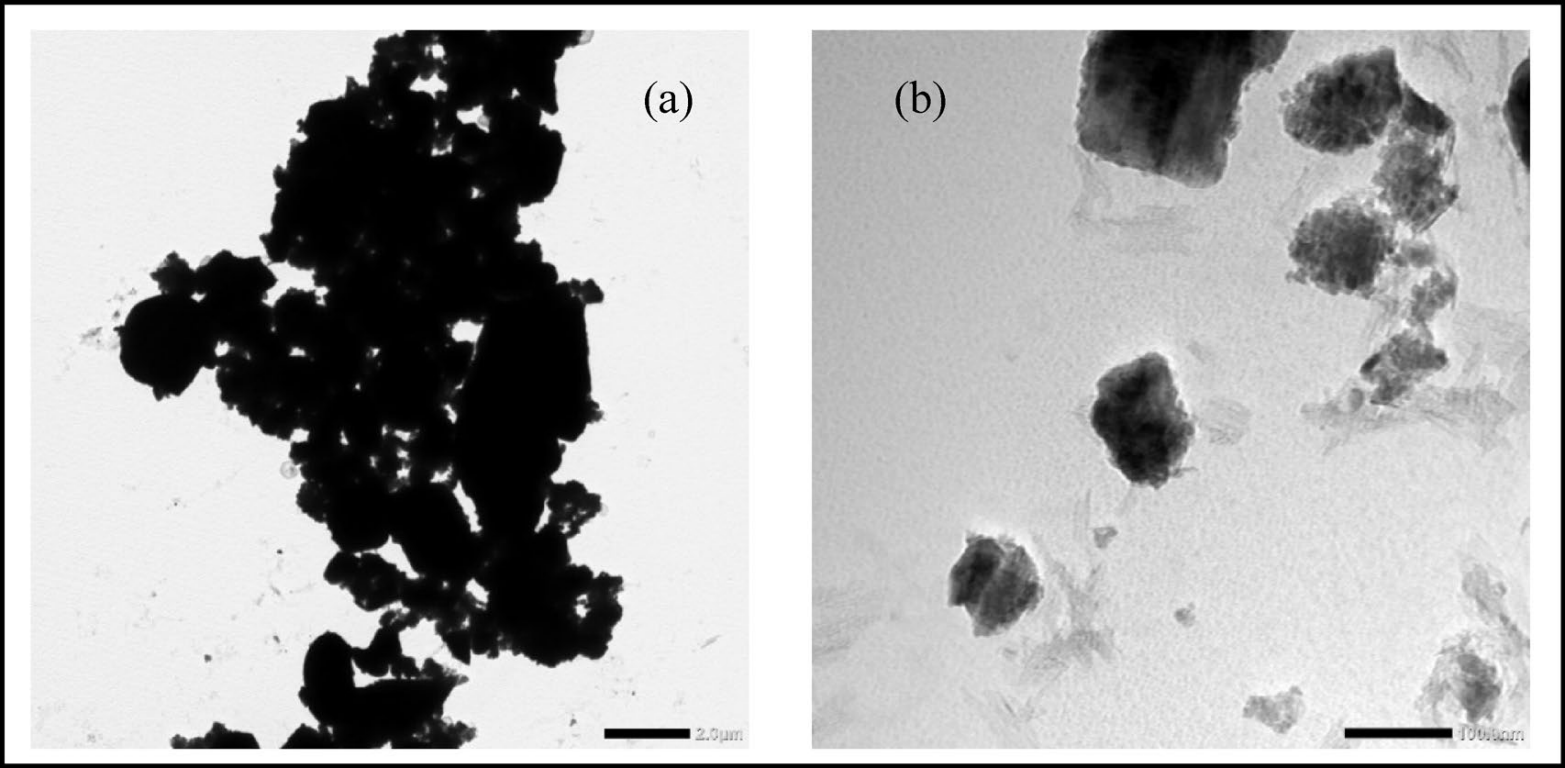
TEM images of (**a**) Al_2_O_3_ microparticles size and (**b**) Al_2_O_3_ nanoparticles size.

And Al_2_O_3_ in the micro scale was in the size of (2$\:\mu\:$m) that is presented in Fig. 5a and after ball milling the oxide it became in the NPs of approximately (50 nm) as shown in Fig. 5b.

### Scanning electron microscope (SEM)

**Fig. 6:**
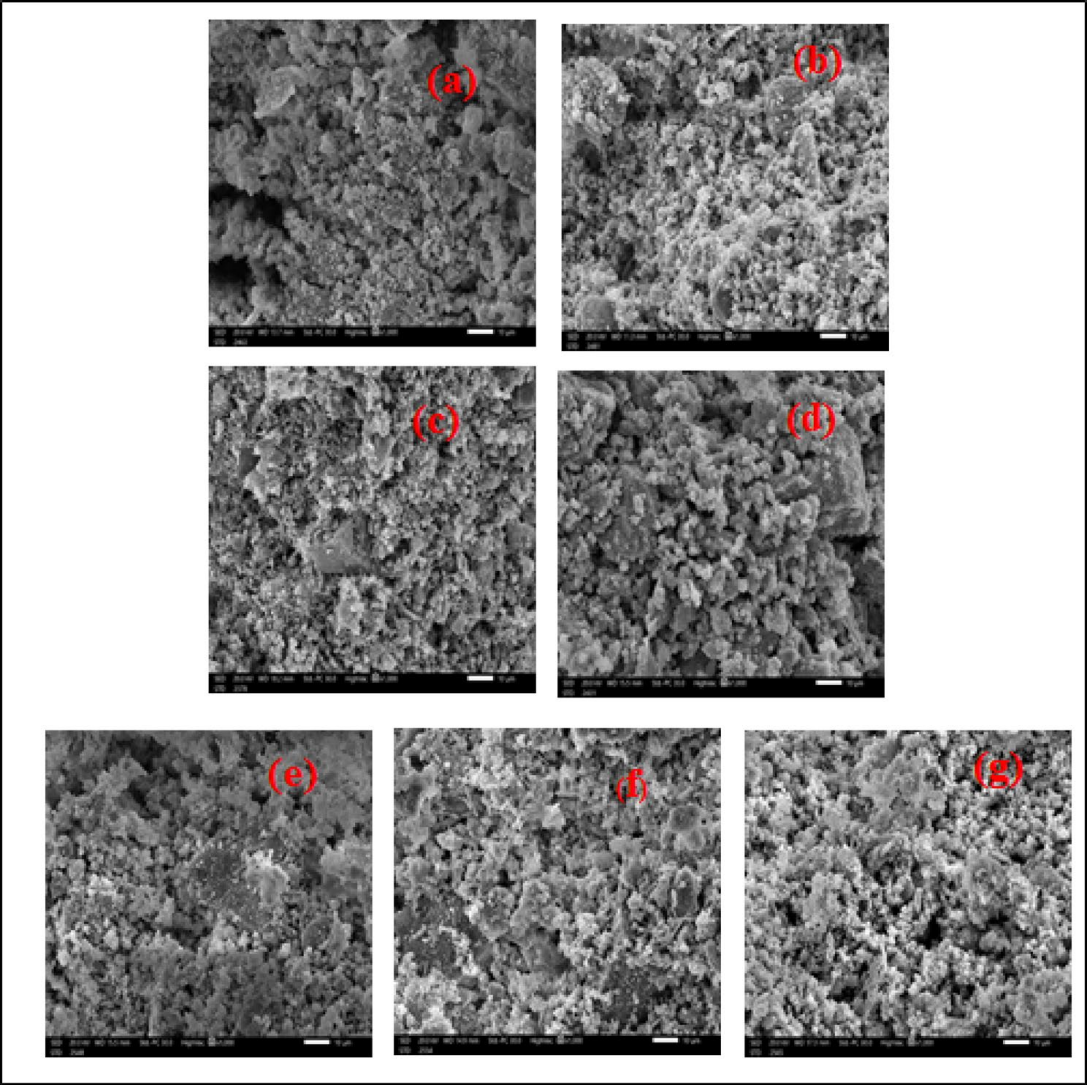
(**a**) SEM images of CM (**b**) SEM images of CM-M1 (**c**) SEM image of CM-M2 (**d**) SEM image of CM-M3 (**e**) SEM images of CM-N1 (**f**) SEM images of CM-N2 (**g**) SEM images of CM-N3.

The morphology of composites with and without fillers in the composites with varied weight percentages and particle sizes of CdO and Al_2_O_3_ is different, as illustrated in Fig. 6. In the case of nano-CdO/Al_2_O_3_ composites, the CdO and Al_2_O_3_ NPs are evenly distributed, which may enhance the interfacial adhesion between cement and CdO/ Al_2_O_3_ NPs as well as offering an interlocking structure for shielding. Large CdO particles, on the other hand, are poorly covered by the cement matrix in micro-CdO/ Al_2_O_3_ composites, and some of them peel away owing to weak interfacial adhesion, creating shielding gaps. Because of the expected better shielding performance of these nanocomposites as seen by the SEM images, the distribution of nanoparticles should be more uniform than that of micro particles.

### Mechanical properties

Because the composites include varying amounts of micro and nano CdO/Al_2_O_3_, it is discovered that the composites stretch evenly in the low strain area, in accordance with Hooke’s law. As micro/nano CdO/ Al_2_O_3_ concentration increases, the initial modulus is improved. Tensile strength and elongation at break are also improved. Nano composites are more resilient to applied compression than micro composites, and the more nano CdO/ Al_2_O_3_ concentration present in the samples, the more brittle they become. Additionally, Table 3 displays the ultimate force, stress, and break distance for the composites. It reveals that the CM-N3 sample had the highest ultimate force value, while the sample with the lowest value was CM-M3.

**Table 3:** Ultimate force, stress, and break distance from mechanical compression.

Parameters	CM	CM-M1	CM-M2	CM-M3	CM-N1	CM-N2	CM-N3
Ultimate force (N)	3792.00	10844.28	2943.33	2450.00	4304.00	10851.66	10993.18
Ultimate stress (MPa)	7.72	22.09	6.00	4.99	8.77	22.11	21.99
Break distance (mm)	4.56	5.28	4.21	3.40	4.05	5.20	6.94

### Shielding parameters

A commonly used metric for analyzing and contrasting the shielding effectiveness of various shielding materials is the mass attenuation coefficient, or$\:\:{{\upmu\:}}_{\text{m}}\left(\text{c}{\text{m}}^{2}{g}^{-1}\right)$. The measured mass attenuation coefficient values derived from Eq. (3), the theoretical mass attenuation coefficient values acquired from the XCOM algorithm, and the measured densities of all composites for energies ranging from 59.53 to 1408.01 keV are listed in Table 4. Add to the list the additional parameters, which are the relative deviation RD (%), which is determined by Eq. (13) and the MFP, HVL, and TVL of the composites, which are determined by Eqs. (6), (4), and (5), respectively:

13 $$\:RD\left(\%\right)=\frac{{\left(\frac{\mu\:}{\rho\:}\right)}_{XCOM}-{\left(\frac{\mu\:}{\rho\:}\right)}_{EXP}}{{\left(\frac{\mu\:}{\rho\:}\right)}_{XCOM}}*100\%\:$$

The $\:\left(\frac{{\upmu\:}}{{\uprho\:}}\right)$ values obtained using the XCOM online database of photon interaction cross-sections relative to the experimental data with γ-ray energy in the range of 0.059–1.408 MeV.

**Fig. 7:**
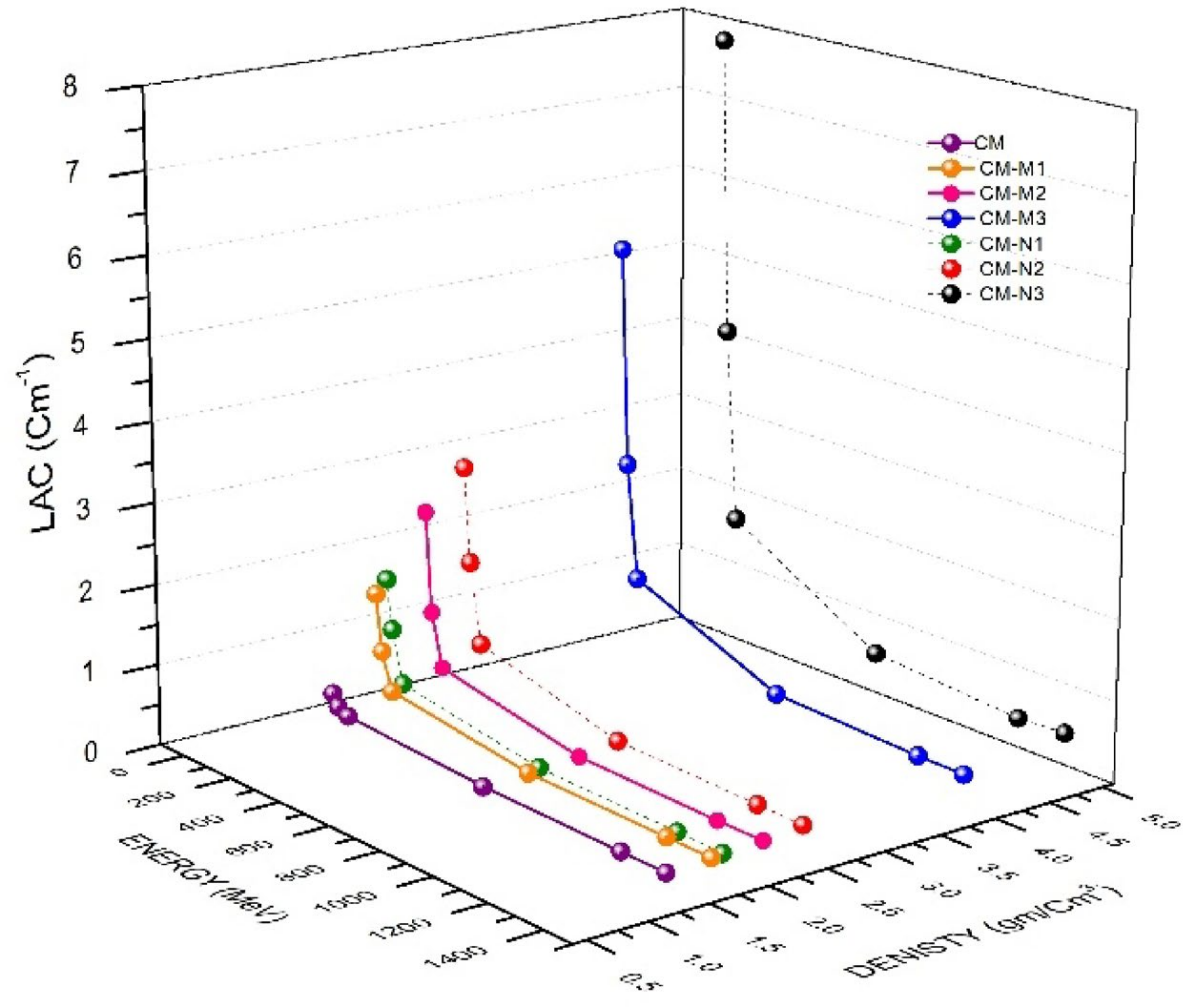
Comparison between linear attenuation coefficient and density for cement/marble (micro, nano) CdO + Al_2_O_3_ composite experimentally.

**Fig. 8:**
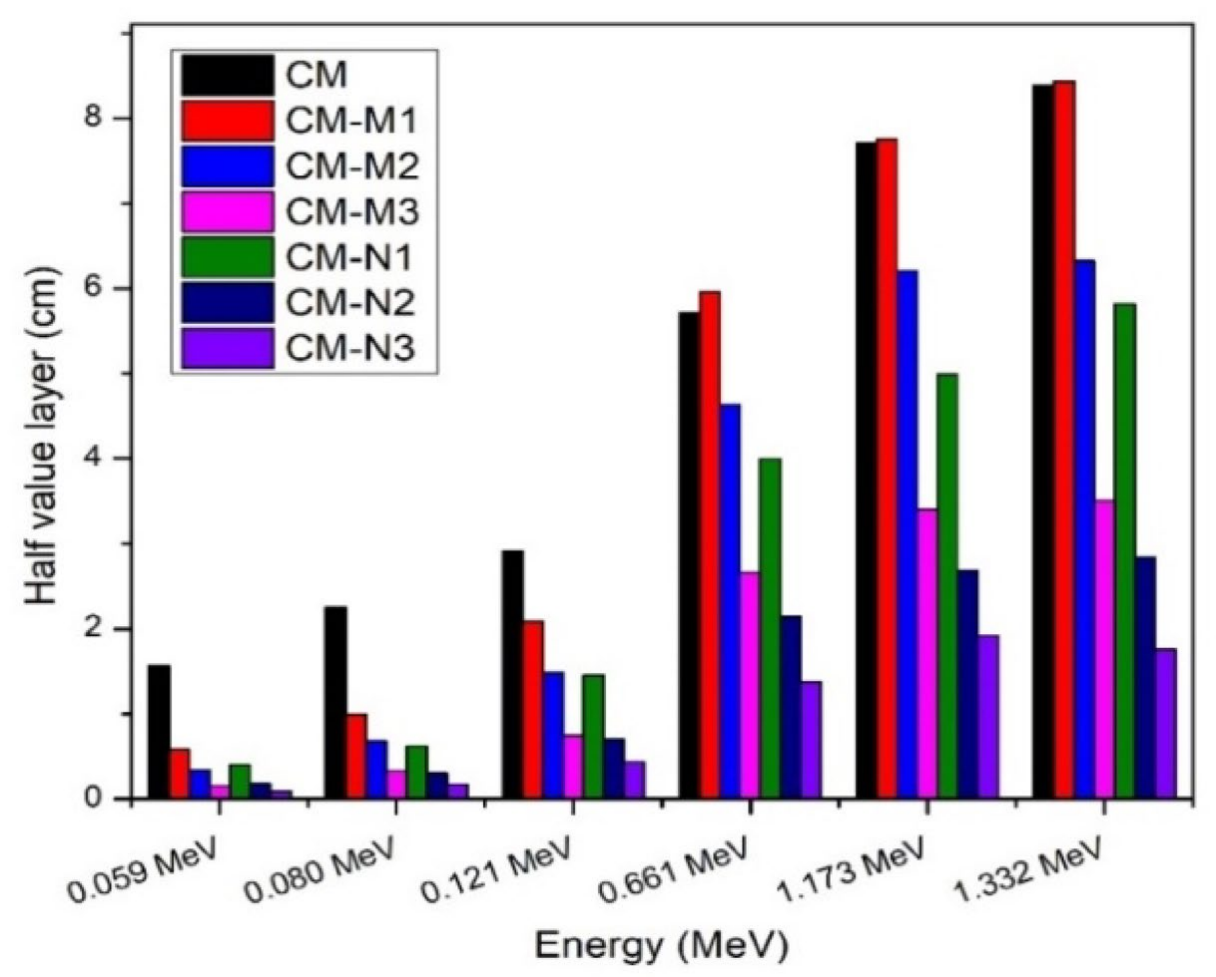
Comparison between half value layer for cement/marble (micro, nano) CdO + Al_2_O_3_ composite experimentally.

**Fig. 9:**
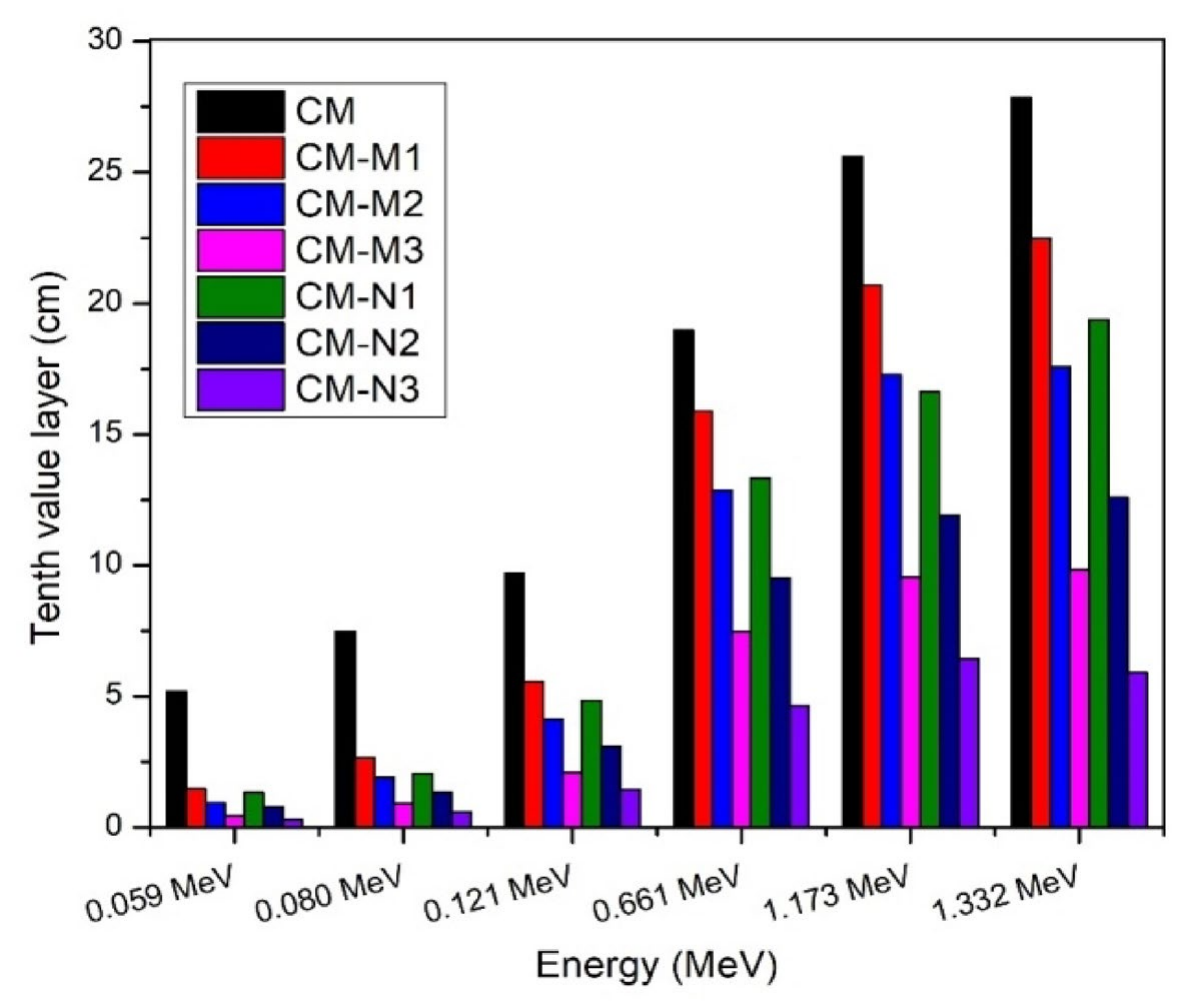
Comparison between tenth value layer for cement/marble (micro, nano) CdO + Al_2_O_3_ composite experimentally.

**Fig. 10:**
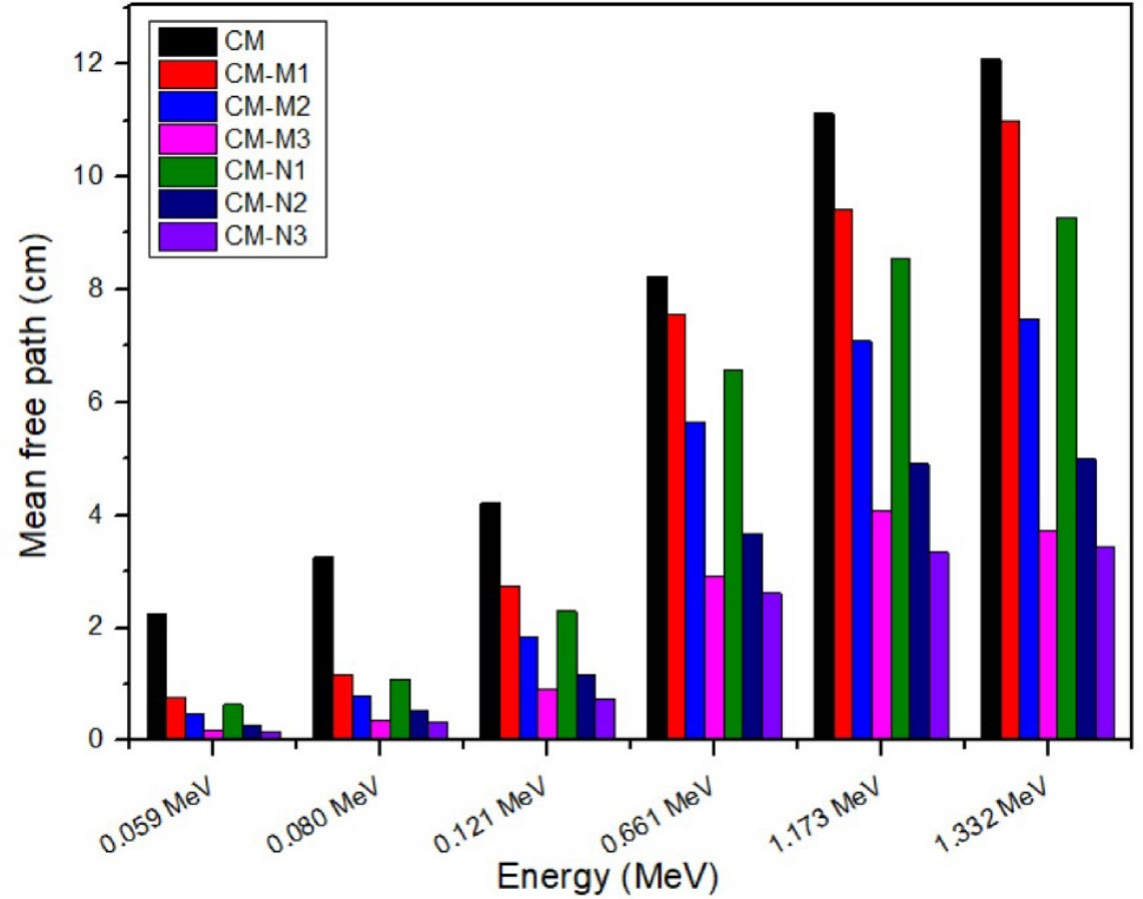
Comparison between mean free path for cement/marble (micro, nano) CdO + Al_2_O_3_ composite experimentally at different energies.

LAC, HVL, TVL and MFP are represented in Figs. 7, 8, 9 and 10 respectively for the energies between (0.015 to 15 MeV) in this study, comparing the linear attenuation coefficient (LAC ) for micro and nano fillers composites, It can observed that the CM-N3 has a greater LAC than CM-M3 as shown in Fig. 7; Table 4 addressed the relationship between increasing surface area per unit volume and decreasing particle size, and outcomes indicate to the feasibility of using nanoparticles to enhance the shielding properties currently used radiation shielding materials, When we add Cement/Marble +(CdO (10%) + Al_2_O_3_(10%)) in micro and Nano particle sizes samples, It can be seen that the CM-N1 has a greater LAC than CM-M1 as shown in Fig. 7; Table 4.

The half-layer value is a very important parameter Fig. 8; Table 4 showing how half-layer value (HVL) for sample CM-M1, CM-M2, CM-M3, CM-N1, CM-N2 and CM-N3 The predicted behavior was that the HLV would drop as more nanomaterial was incorporated into the shielding material. This reduction in HLV was more pronounced at high energy than low energy, indicating that the HVL would decrease at the same energy as the concentration of nano CdO increased.

One crucial component is the value within the tenth layer. CM-M (1–3) and CM-N (1–3) samples’ tenth-layer values are displayed in Fig. 9; Table 4. TLV decreased by adding nanoparticles to the shielding material. The TVL decreased more at high energy than at low energy, indicating that the TVL would also decrease at the same energy with an increase in Nano CdO concentration.

The mean free path is the average distance a photon travels through an absorber before removal from the initial beam via absorption or scattering. And it is a very important characteristic of shielding material. Figure 10; Table 4 shows the MFP for sample CM-M-M (1–3) and CM-N (1–3) MFP decreased by incorporating Nano material to the shielding material, this reduction in MFP becomes more distinct at high energy than low energy.

When we compare MACs between (Micro and Nano) Cement/Marble + CdO/Al_2_O_3_ composites at different concentration with MACs of (micro and nano) Cement /Marble + Iron slag^8^ and then calculate relative difference between two different composites as shown in Table 5^8^, It can be seen that at 0.059 MeV the MACs of CM-micro (CdO/Al_2_O_3_) composites is higher than MACs of CM-MIS composite. But at higher energies the MAC of CM-micro (CdO/Al_2_O_3_) comes very near the values that of the micro iron slag CM composite. So, the significant variation in the MAC values for both MPs and NPs CM + CdO/Al_2_O_3_ are clear at low energies from (0.059 to 0.121 MeV), but as the energies increases that variation comes to be nearly equal^18^.

**Fig. 11:**
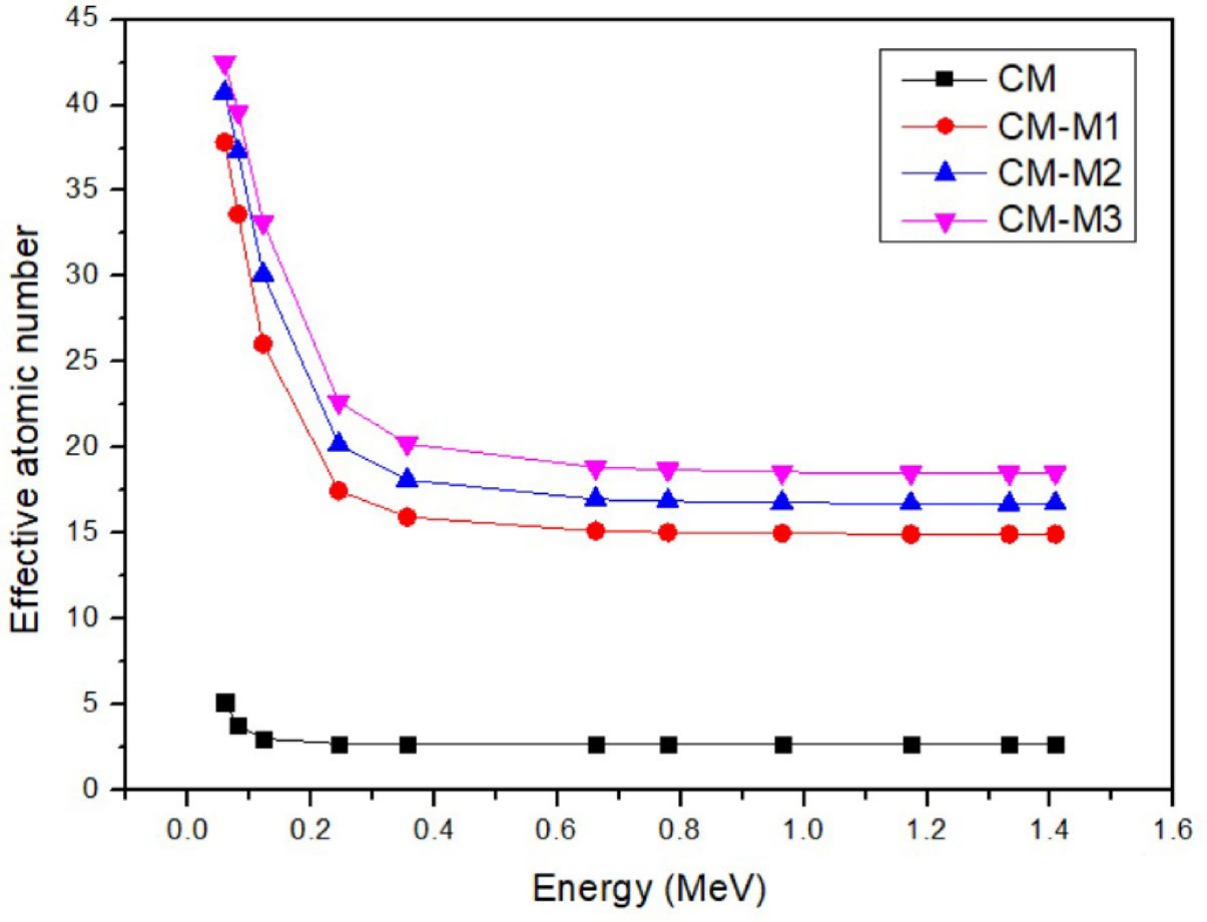
Effective atomic no. vs. photon energy.

To get the buildup factor, the effective and equivalent atomic numbers also need to be computed. Figure 11’s effective atomic number shows how the high atomic numbers of the Cd and Al elements lead the concentration of CdO and AL_2_O_3_ in the cement + marble composite to grow when the filler weight% increases.

**Fig. 12:**
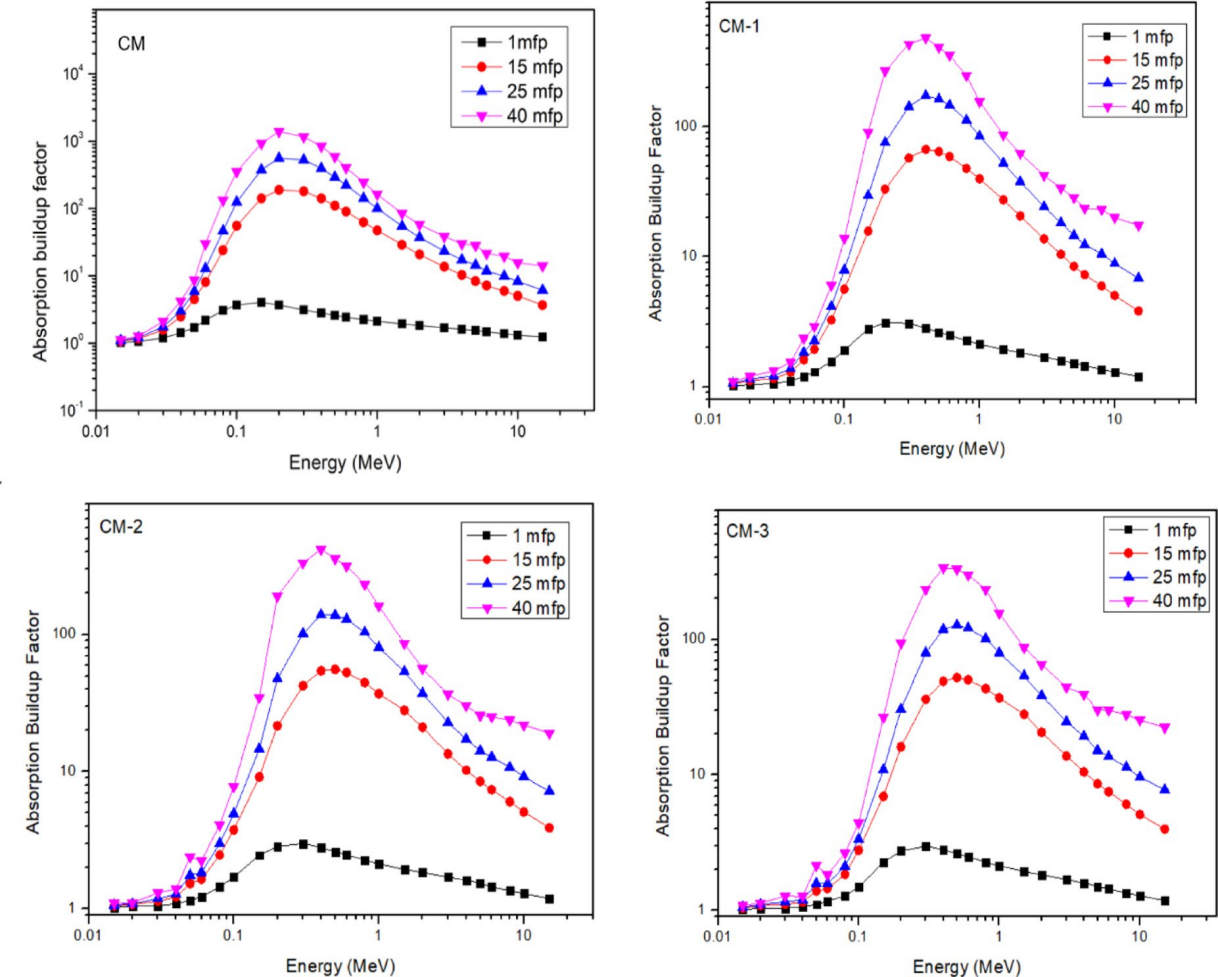
Absorption buildup factor with gamma ray energy for cement/marble/CdO + Al_2_O_3_ composite.

Figure 12 shows the fluctuation of the accumulation factor at penetration depths of 1, 15, 25, and 40 mfp with photon energy ranging from 0.015 to 15 MeV. Figure 12 makes it evident that all composites have low EABFs in the low energy zone and that these values only marginally rise when the penetration depth is increased to 40 mfp. These low EABF values might be caused by the photoelectric effects, in which at low photon energy all photons are completely ejected from the material. Put another way, photon interactions with the sample increase with penetration distance, leading to a commensurate rise in the number of low-energy photons created^43^.

Additionally, the highest EABF is seen in the middle energy range, this is thought to be a common pattern for all of the CdO composites that have been studied. The reason for this is that the photon undergoes repeated scattering during the Compton scattering process, which reduces its energy but leaves it partially intact. As a result, the EABF reaches a maximum value of 0.21–1 MeV. This is explained by the fact that higher Cd element concentrations enhance the attenuation likelihood of photons, which is in line with earlier findings. Therefore, the pair creation process combined with the annihilation process doubles the photons instead of absorbing extremely energy photons (over 5 MeV). It is demonstrated that the EABF values are quite high for deep penetration and in the pair production zone.

Increases in the penetration depth of the materials for the material with the greatest equivalent atomic number result in an increase in the thickness of the interacting material, which in turn produces an increase in the number of scattering events in the interacting medium.

**Table 4:** Measured values of MACs, RD%, MFP, HAVL, TVL, and density of different concentrations of composites.

Sample	Energy (MeV)	Mass attenuation coefficient (cm^2^/g)		RD%	HVL (cm)	TVL (cm)	MFP (cm)	Density (g/cm^3^)
		EXP	X-COM					
CM	0.059	0.2890	0.2875	− 0.5252	1.5604	5.1836	2.2512	1.537$\:\pm\:$0.102
	0.080	0.2002	0.1998	− 0.2052	2.2525	7.4827	3.2497	
	0.121	0.1546	0.1522	− 1.5966	2.9165	9.6883	4.2076	
	0.661	0.0789	0.0769	− 2.6743	5.7132	18.9787	8.2423	
	1.173	0.0585	0.0586	0.1366	7.7090	25.6086	11.1217	
	1.332	0.0538	0.0549	2.0033	8.3809	27.8406	12.0910	
CM-M1	0.059	0.7762	0.7774	− 0.1544	0.5780	1.9201	0.8339	1.545$\:\pm\:$0.054
	0.080	0.4512	0.4034	11.8493	0.9943	3.3031	1.4345	
	0.121	0.2153	0.2134	0.8997	2.0836	6.9215	3.0060	
	0.661	0.0753	0.0763	− 1.3103	5.9564	19.7869	8.5933	
	1.173	0.0578	0.0579	− 0.0812	7.7588	25.7743	11.1936	
	1.332	0.0532	0.0542	− 1.8916	8.4308	28.0067	12.1631	
CM-M2	0.059	1.0364	1.0220	1.4090	0.3375	1.1211	0.4869	1.982$\:\pm\:$0.033
	0.080	0.5121	0.5051	1.3859	0.6830	2.2689	0.9854	
	0.121	0.2356	0.2440	− 3.4426	1.4846	4.9318	2.1418	
	0.661	0.0755	0.0760	− 0.6839	4.6315	15.3856	6.6819	
	1.173	0.0563	0.0575	− 2.0845	6.2104	20.6304	8.9597	
	1.332	0.0553	0.0539	2.6173	6.3226	21.0033	9.1216	
CM-M3	0.059	1.2710	1.2670	0.3165	0.1554	0.5163	0.2242	3.509$\:\pm\:$0.052
	0.080	0.6115	0.6069	0.7645	0.3230	1.0730	0.4660	
	0.121	0.2645	0.2746	− 3.6708	0.7468	2.4807	1.0774	
	0.661	0.0742	0.0758	− 2.0116	2.6609	8.8393	3.8389	
	1.173	0.0579	0.0572	1.3355	3.4097	11.3267	4.9191	
	1.332	0.0563	0.0536	5.1306	3.5068	11.6493	5.0592	
CM-N1	0.059	1.7279			0.4012	1.3326	0.5788	2.024$\:\pm\:$0.028
	0.080	1.1180			0.6200	2.0596	0.8945	
	0.121	0.4773			1.4523	4.8243	2.0952	
	0.661	0.1733			3.9990	13.2842	5.7693	
	1.173	0.1389			4.9895	16.5748	7.1983	
	1.332	0.1192			5.8138	19.3129	8.3875	
CM-N2	0.059	1.0739			0.1784	0.5925	0.2573	3.162$\:\pm\:$0.027
	0.080	0.6410			0.2988	0.9925	0.4311	
	0.121	0.2735			0.7002	2.3260	1.0102	
	0.661	0.0895			2.1410	7.1123	3.0889	
	1.173	0.0712			2.6885	8.9309	3.8786	
	1.332	0.0674			2.8400	9.4343	4.0972	
CM-N3	0.059	1.5101			0.0890	0.2956	0.1284	5.159$\:\pm\:$0.021
	0.080	0.7838			0.1714	0.5694	0.2473	
	0.121	0.3102			0.4331	1.4388	0.6249	
	0.661	0.0976			1.3772	4.5750	1.9869	
	1.173	0.0699			1.9219	6.3843	2.7727	
	1.332	0.0763			1.7603	5.8476	2.5396	

This study successfully developed Cement + Marble + CdO/Al₂O₃ composites as enhanced gamma-radiation-shielding building materials, demonstrating that nano-sized CdO/Al₂O₃ particles significantly outperform micro-sized particles in shielding effectiveness across all tested energy levels. The experimental results showed excellent agreement with theoretical XCOM database values, with nanocomposites exhibiting higher mass attenuation coefficients (MAC) and lower half value layer (HVL), tenth value layer (TVL), and mean free path (MFP) values compared to micro-composites. With optimal concentrations below 25% to maintain material integrity while maximizing shielding capability. These findings provide crucial insights for developing advanced radiation-shielding materials with applications in nuclear engineering and medical physics.

## Simulations

FLUKA, a widely used Monte Carlo code, has given significant success in simulating the attenuation of photons and neutrons across a wide range of materials. Its accurate physics models and cross-section libraries, like ENDF, enable success predictions of radiation transport and interaction processes. This code is one of the most practical simulation tools for many applications in high-neutron physics and engineering aspects^44^.

Recently, it has been shown that FLUKA and MCNP are an appropriate Monte Carlo simulation code to investigate the radiation shielding properties of various glass and cement systems^43–45^. FLUKA, a widely used Monte Carlo code, has given significant success in simulating the attenuation of photons and neutrons across a wide range of materials. Its accurate physics models and cross-section libraries, like ENDF, enable success predictions of radiation transport and interaction processes. This code is one of the most practical simulation tools for many shielding applications in high-neutron physics and engineering aspects. FLUKA can simulate the interaction and propagation in matter of more than 60 different particles, such as heavy-ions, electrons, neutrons, photons, neutrinos and muons, in many types of research fields: shielding design, radiation detector response studies, medical physics and dosimetry calculations. After permissions the code was installed and run under Ubuntu 20.04.6 LTS (Focal Fossa) operating system.

**Fig. 13:**
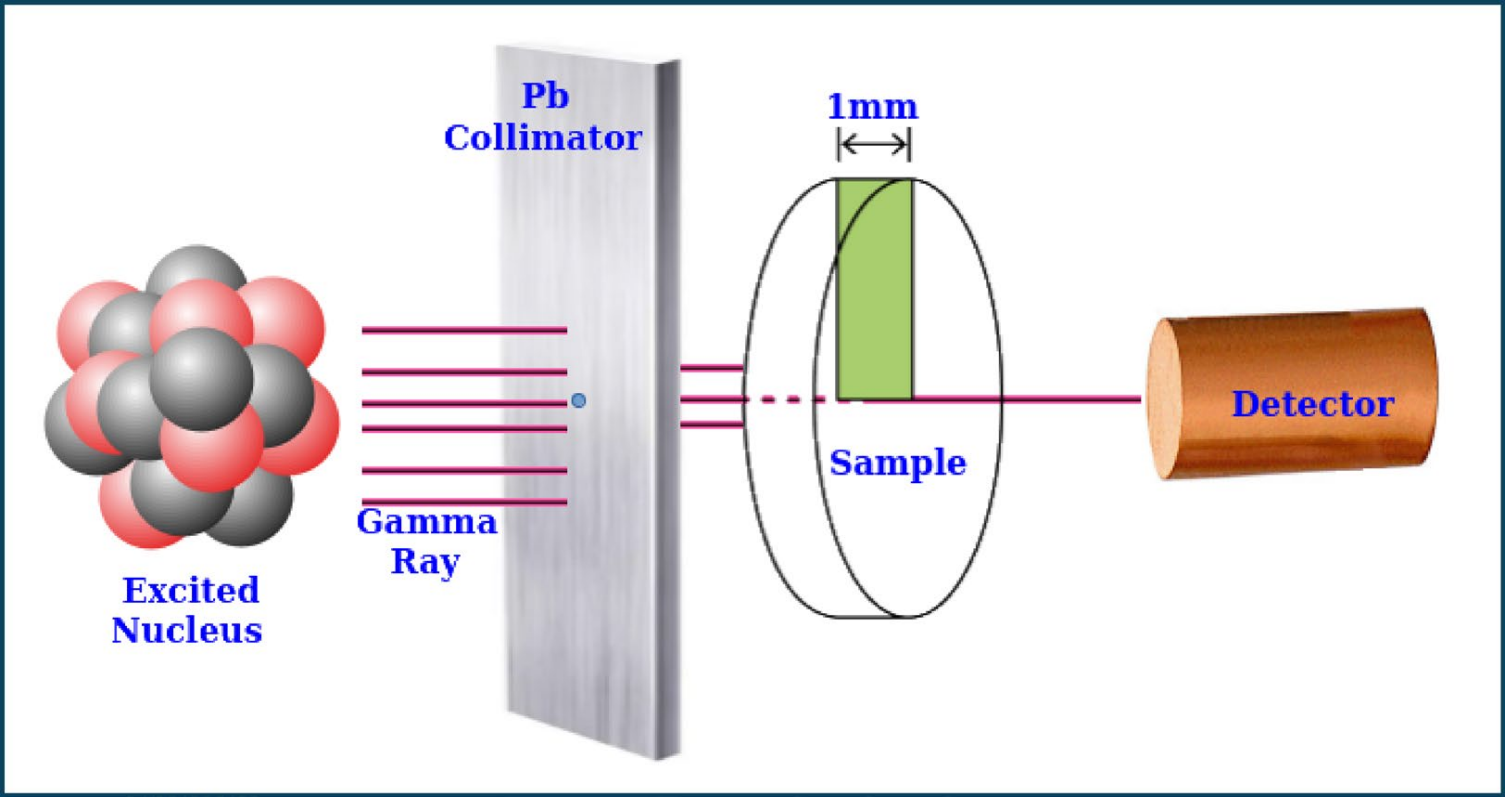
Systematic attenuation system used in the simulation.

In this study, a general-purpose FLUKA code was used to obtain the shielding capability of the different introduced composites. The used open-source software FLUKA version was (4-1.1) was used with its advanced graphical user interface FLAIR version (3.1–15.1)^46,47^. The FLAIR Interface allows us to interact easily with FLUKA projects. In the present simulation, mono-energetic gamma ray energy (0.01-100 MeV) and (0–20 MeV) mono energetic neutron was adjusted. The beam characteristics (direction of the source) are defined in BEAM and BEAMPOS input cards. A rectangular box with 5 cm height, 5 cm width, and 0.1 cm thickness has been chosen for the target shape geometry. Samples have been described using the built-in MATERIAL and COMPOUND cards using Ca, O, Si, Al, S, Fe, Mg, Ti, C and Cd built in elements. In the case of natural elements, the 87 ENDF/B/VIIIRO library was recommended^21,25^. Simulations were done for 10^6^ primary particles, and the code was run for 5 cycles. The results were directed at output binary files of USRBIN and USRBDX. The average and mean values were calculated using the FLAIR RUN mode. The simulated explanation is represented in Fig. 13 and appendix A.

**Fig. 14:**
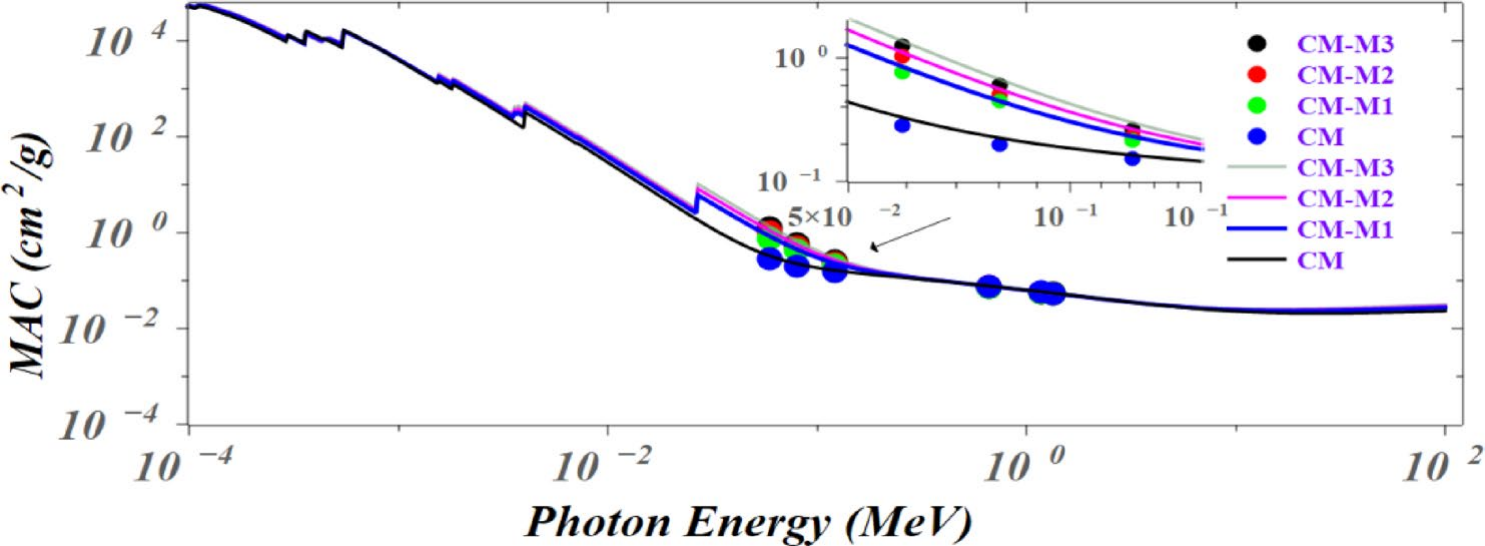
Results of simulation of mass attenuation coefficient MCA for a wide photon energy range, the filled circle points refer to the experimental data.

**Fig. 15:**
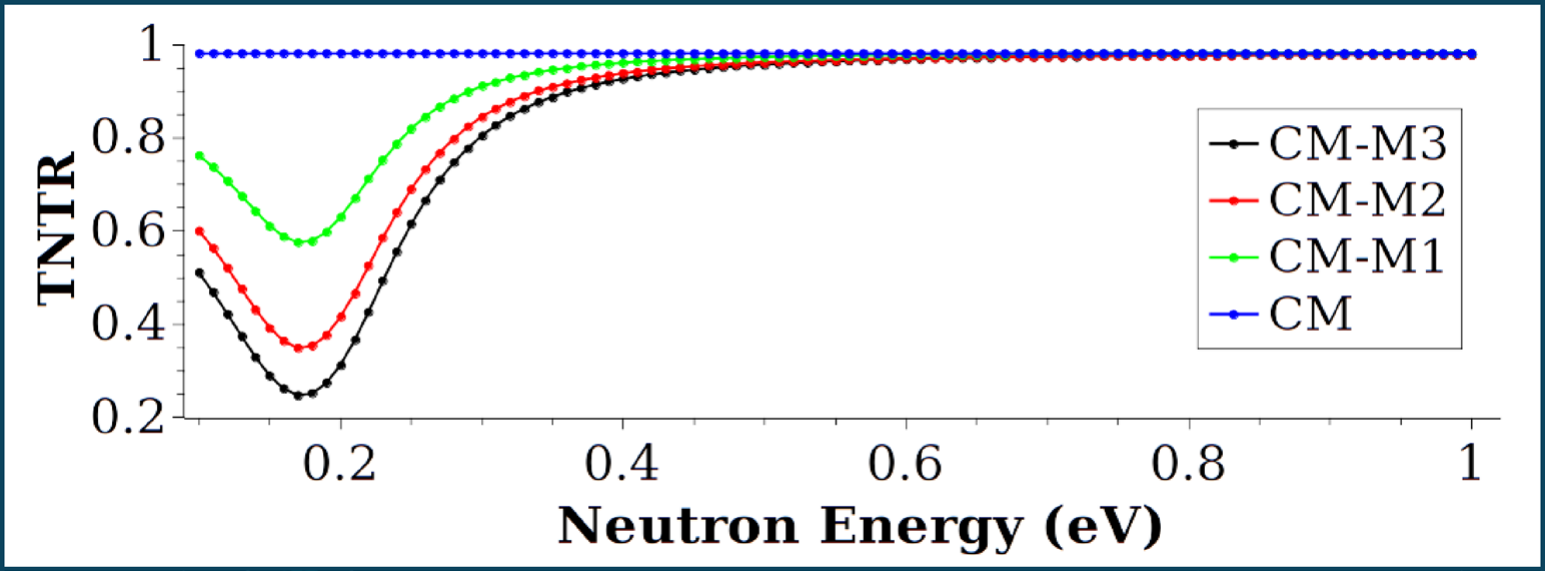
Neutron transmission ratio TNTR vs. thermal neutron energy.

The FLUKA software simulation was used to calculate the mass attenuation coefficient over a wide energy range of 0.1 keV to 100 MeV. The results, with comparison with experimental data, are presented in Fig. 14. As observed in Fig. 15, distinct sharp peaks are present at 0.0003, 0.0004, 0.0005, 0.0006, 0.0015, 0.0018, 0.0036, 0.0041, and 0.026 MeV. These sharp peaks observed in the mass attenuation curve are primarily due to the photoelectric effect. This effect dominates the lower photon energies and involves the complete absorption of a photon by an atom, ejecting an inner-shell electron. Furthermore, the curves overlap in several regions, particularly at higher photon energy^48^.

**Fig. 16:**
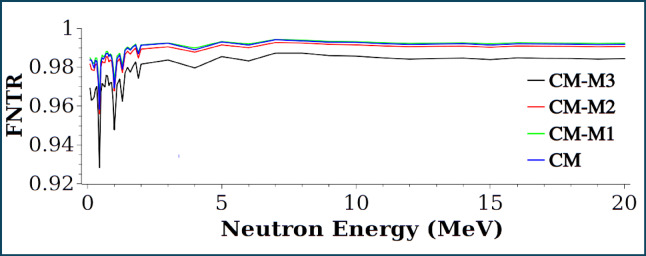
Neutron transmission ratio (FNTR) vs. fast neutron energy.

Figures 15 and 16 represent thermal and fast neutron transmission ratios (TNTR, FNTR) for samples CM, CM-M1, CM-M2 and CM-M3. In terms of fast neutron transmission, sample CM-M3 demonstrates superior performance with bottom values reaching up to 2 MeV. It should be mentioned that neutrons released during fission process can be considered fast neutrons with an average energy of 2 MeV. As a result, CM-M3 samples can be viewed as excellent shielding material for reactor buildings. For thermal neutron transmission curves diverge below 0.4 eV due to differences in the energy-dependent scattering cross-sections of the materials^49^.

**Fig. 17:**
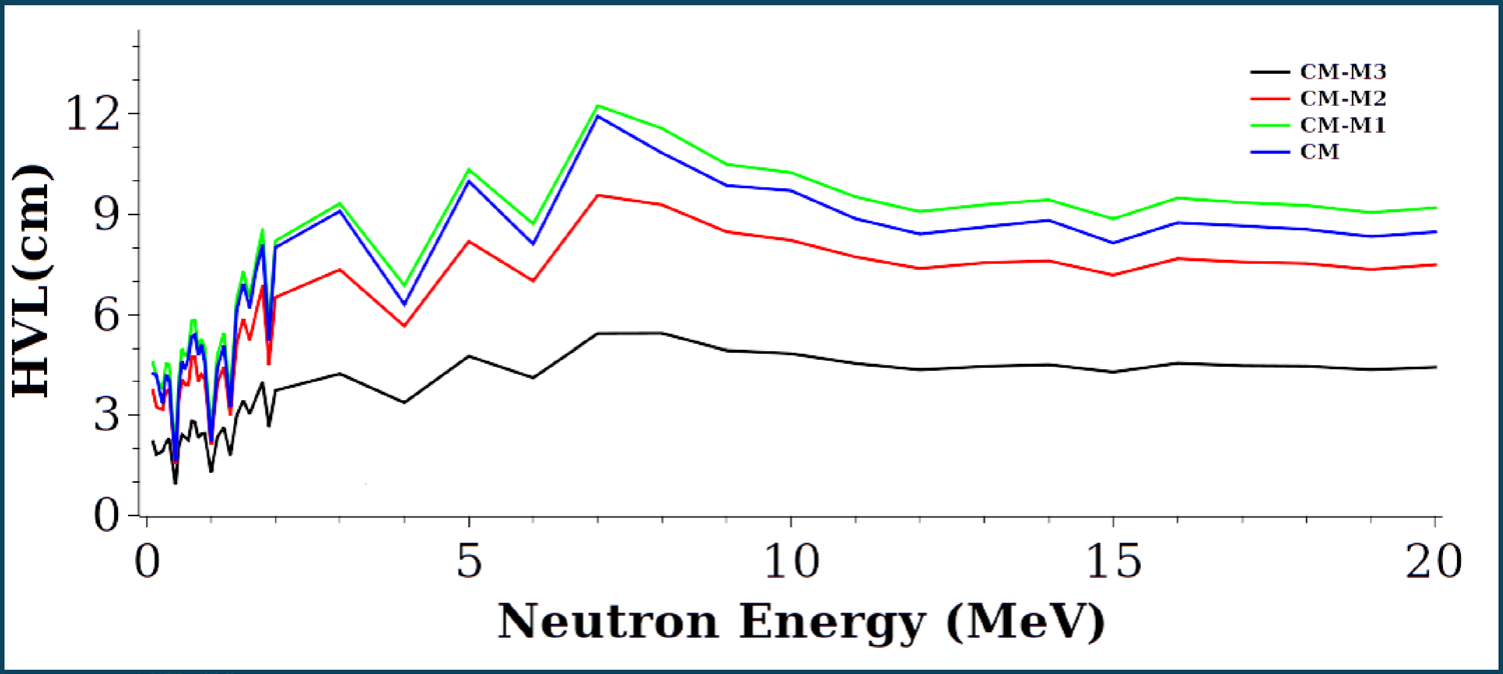
Removal cross section Σr for 0–20 MeV neutron energy range.

**Fig. 18:**
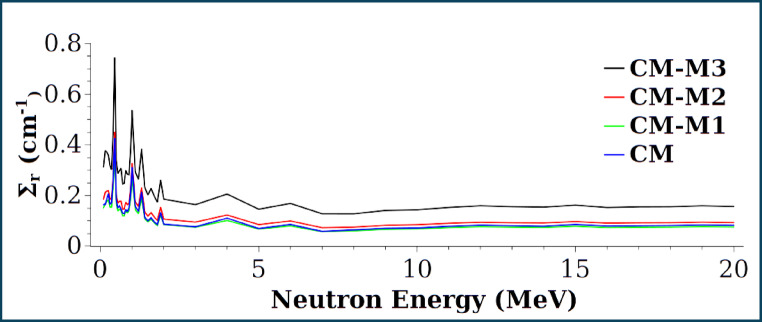
Half value layer HVL for neutron energy up to 20 MeV.

Figures 17 and 18 show the removal cross-sections and half-value layers of the CM, CM-M1, CM-M2, and CM-M3 composites for neutron energies up to 20 MeV. CM-M3 has the highest Σr values, with peaks near 0.44, 1.0, 1.34, and 1.9 MeV, likely due to resonance scattering, resonance absorption, or inelastic scattering. CM-M3 also offers the best shielding performance, as indicated by its lowest HVL^50^.

The experimental half-value layer (HVL) of paraffin wax^51^, which is one of the most effective neutron absorbers, was compared with our calculated values for 4.5 MeV neutrons, as shown in Table 5. It was found that sample CM-M3 closely approximates the properties of paraffin wax, suggesting that it could be considered an effective neutron shield.

**Table 5:** HVL values of samples against neutrons with 4.5 MeV.

Shielding material	FLUKA HVL (cm)
Paraffin wax	3.96
CM	8.6
CM-M1	8.1
CM-M2	6.9
CM-M3	4.01

## Conclusion

The outcome of this study shows that after fabricating Cement + Marble + CdO/Al₂O₃ composites with various concentrations to develop new building materials with improved gamma-radiation-shielding capabilities, we observed that when the concentration of micro CdO/Al₂O₃ reached 25% or more, the samples became brittle while the nano sample for the same concentration become more ductile.

There was good agreement between the experimental values of the mass attenuation coefficient (MAC) for micro composites and those obtained theoretically from the XCOM database. The experimental results indicated that the size and concentration of CdO/Al₂O₃ particles significantly influenced the gamma radiation shielding effectiveness of ball clay and cement across all tested energy levels.

Scanning Electron Microscopy (SEM) analysis revealed that the distribution of nano-sized CdO/Al₂O₃ was more uniform compared to micro-sized CdO particles. Nano particles, with their higher electron density, result in a greater interaction probability between photons and CdO, thereby enhancing the shielding capability.

In comparison to Cement and Marble with micro-CdO, the MAC for the nano-CdO/Al_2_O_3_ composites was higher. This suggests that using nanoparticles instead of microparticles improves the shielding capacity of the materials. Additionally, the half value layer (HVL) and tenth value layer (TVL) as well as the mean free path (MFP) values were lower for nanocomposites compared to micro-composites, indicating that nanocomposites are more effective at attenuating gamma rays.

The FLUKA simulations show the effectiveness of the introduced composites in attenuating both gamma rays and neutrons. The mass attenuation coefficients for photons presented distinct peaks due to the photoelectric effect on lower energies, while the neutron transmission ratios highlighted the higher performance of certain composites in reducing both thermal and fast neutron fluxes. The study also investigated the removal cross-sections and half-value layers for neutron energies up to 20 MeV, demonstrating the potential of specific composites for neutron shielding applications. These composites show promising potential for use in patient safety and radiation protection within medical settings such as hospitals. However, further biocompatibility and dosimetry studies will be essential before their direct application in clinical practice.

### Future work

Future studies will focus on the development of multi-layer shielding systems by combining composites with other materials, exploring graded nano/micro-filler layers, and incorporating fiber reinforcement to improve toughness. Dose-mapping experiments under different radiation conditions will also be conducted to verify in-situ performance.

## Supplementary Information

Below is the link to the electronic supplementary material.

Supplementary Material 1

## Data Availability

The datasets used and/or analysed during the current study available from the corresponding author on reasonable request.
