# Supplementary material for: Experimental and FLUKA simulation study of CdO /Al2O3 cement waste marble composites for nuclear radiation shielding
Source: Sci Rep. 2025 Oct 8;15:35043. doi: 10.1038/s41598-025-21061-y (PMC12508465; doi:10.1038/s41598-025-21061-y)
Supplement: Supplementary file 1 — Supplementary Material 1 [file 41598_2025_21061_MOESM1_ESM.pdf]

Appendix (A): SECTION WITH KEY EXCERPTS FROM THE USED FLUKA INPUT DECK DECLARED USING FLAIR MODE

1- Simulation Setup and Incident Beam Adjustments

```
#define  ENERGY        : 10
..+....1....+....2....+....3....+....4....+....5....+....6....+....7....+....8
BEAM                 Beam: Energy ▾      E: =-ENERGY  Part: NEUTRON ▾
         Δp: Flat ▾      Δp:             Δϕ: Flat ▾       Δϕ:
 Shape(X): Rectangular ▾ Δx:       Shape(Y): Rectangular ▾ Δy:
DEFAULTS                      : PRECISIO ▾
BEAMPOS                 x: 0.0          y: 0.0          z: -50.
                     cosx:           cosy:           Type: POSITIVE ▾
```

The #define card is used to adjust the energy values to looping. This loop is adjusted to create a selected simulation runs. It iterates through different energy values for both neutrons and photons.

For neutrons, the loop runs within a defined of 50 times. For photons, the loop runs within a defined energy range of 200 times. The Number 10 in the define energy card is optional and does not affect any of the calculations the default is -.

The Beam card defines the mono-energetic primary particle, loop for photons and neutron. Particles are selected as neutron or photon. Negative sign denotes to specify kinetic energy. BEAMPOS: Sets the beam's starting position at (0, 0, −50)

2- Geometry Definition

```
GEOBEGIN      Accuracy:             Option: ▾            Paren:
              Geometry: ▾              Out: ▾              Fmt: COMBNAME ▾
      Title: A simple Glass target inside vacuum
        RPP  extvoid Xmin: -200.        Xmax: 200.
                     Ymin: -200.        Ymax: 200.
                     Zmin: -200.        Zmax: 200.
        RPP  invoid  Xmin: -100.        Xmax: 100.
                     Ymin: -100.        Ymax: 100.
                     Zmin: -100.        Zmax: 100.
        RPP  targ    Xmin: -10.         Xmax: 10.
                     Ymin: -10.         Ymax: 10.
                     Zmin: 0.0          Zmax: 0.1
        XYP  p1          z: 0.0
        plane to separate the right side of target
        XYP  p2          z: 0.1
END
```

- The outer most boundary of the simulation, a large box named extvoid, extending from −200 to 200 cm in all three dimensions (x, y, z).
- The invoid defines a smaller inner void that will contain the core of the setup.
- The target sample. It's a thin slab with a thickness of 0.1 cm along the z-axis, and a square cross-section of 20×20 cm.
- p1 is the plane at z=0, and p2 is the plane at z=0.1. These planes are used to define regions around the target.

3- Sample Preparation Protocols

- Target region defined as a rectangular parallelepiped with dimensions 0.2 cm × 0.2 cm × 0.1 cm in (x, y, z) directions with target thickness of 0.1 cm.

4- Regions

- The black hole region (bh) is the space inside extvoid but outside invoid.
- The vacuum region before the target is the space inside invoid.
- The target region is defines as the actual sample region.

5- Using ENDF-III0 Evaluated Nuclear Data Library

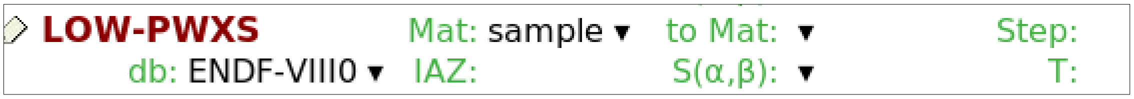

The LOW-PWXS card is used to load the ENDF-VIII.0 library for the required materials. This library provides detailed cross-section data for low-energy neutrons. This is strongly needed for accurately simulating neutron interactions such as elastic scattering, inelastic scattering, and capture reactions.

6- Material Assignments

ASSIGN is the FLUKA card used to assign materials to regions.

BLCKHOLE is assigned to region bh.

VACUUM is assigned to regions valeft and varigh.

The sample material is assigned to the target region.

7- Scoring Cards (USRBDX)

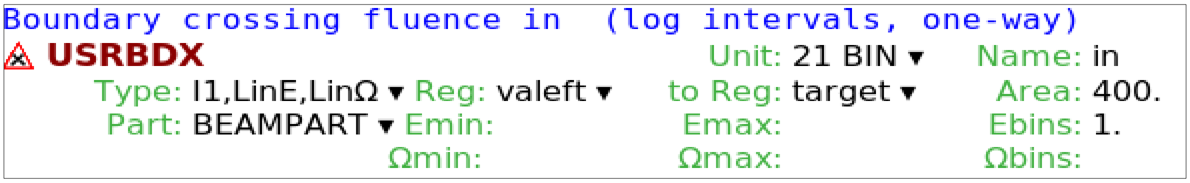

USRBDX cards are used to score the boundary crossing fluence.

• Influence scores the fluence of particles entering the target, specifically neutrons (BEAMPART with type -21).

• Out_fluence scores the fluence of all particles leaving the target and entering the varigh region, providing the transmitted fluence (BEAMPART with type -22).
The beam part can be selected as neutron or photon mode.

8. Statistical Uncertainty

• The simulation was performed with a sufficient number of primary particles to ensure the statistical error on the key results was below 1%.

• A total of 5×10^6 primary particles were simulated, which resulted in a statistical uncertainty of approximately 0.7- 0.8% on the simulated fluence.
